# Factors influencing prey capture success and profitability in Australasian gannets (*Morus serrator*)

**DOI:** 10.1242/bio.047514

**Published:** 2020-01-24

**Authors:** Thomas Cansse, Louarn Fauchet, Melanie R. Wells, John P. Y. Arnould

**Affiliations:** School of Life and Environmental Sciences, Deakin University, 221 Burwood Hwy, Burwood, Victoria 3125, Australia

**Keywords:** Foraging efficiency, *Morus serrator*, Camera, Foraging ecology, Bass Strait, Seabirds

## Abstract

Knowledge of the factors influencing foraging efficiency in top predators can provide insights into the effects of environmental variability on their populations. Seabirds are important marine predators foraging in a highly temporally and spatially variable environment. While numerous studies have focussed on search time and its effects on foraging energetics in seabirds, relatively little is known about the factors influencing capture success and prey profitability in these predators. In the present study, animal-borne cameras were used to investigate the chase durations, capture success, handling durations and profitability of prey consumed by Australasian gannets (*Morus serrator*) (*n*=95) from two breeding colonies in south-eastern Australia exposed to different oceanographic conditions. Capture success was generally lower when individuals foraged alone. However, foraging in multi-species groups and in high prey densities increased chase time, while larger prey elicited longer handling times. While prey type influenced profitability, high prey density and foraging in multi-species groups was found to lower prey profitability due to increased time expenditure. While previous studies have found group foraging reduces search time, the increased profitability explains why some animals may favour solitary foraging. Therefore, future studies should combine search time and the currently found factors.

## Abstract (precis)

**Summary:** In this study, bird-borne cameras were used to investigate the factors influencing capture success, chase and handling time, and prey profitability in Australasian gannets.

## INTRODUCTION

Foraging is a key aspect in the life history of all heterotrophic organisms. Efficient foragers aim to maximize energy intake while keeping the cost of obtaining food to a minimum (Schoener, 1971; Stephens et al., 2008). To do this, foragers have to make choices such as where and when to forage, how long to spend in a food patch and the time spent on handling food. To be profitable, the energy gained by consuming food should be higher than the energy and time spent on finding, handling and consuming food (MacArthur and Pianka, 1966). Profitability can be calculated as the net energy gain divided by the time spent on handling the food and can be used as a measure of foraging efficiency. A higher profitability leads to a higher foraging efficiency and, ultimately, more efficient foragers can be expected to have a higher reproductive fitness and, thus, a higher probability of passing on their genes to the next generation (Pyke, 1984).

Key factors in determining prey profitability are the energetic content of the prey and the energetic cost of obtaining the prey. To increase profitability, predators can target prey with a high nutritional content (Stephens et al., 2008). However, they can also increase profitability by reducing the energetic cost of obtaining the prey. The two main factors influencing the energetic costs of foraging are the time spent searching and the time spent handling food. For pursuit predators, handling time is the time needed to consume the prey and is preceded by a chase phase, which is the time spent catching the prey. One of the ways to optimize foraging efficiency is by reducing the search time, which is the time spent on finding prey. This can be achieved by group foraging, and relying on information obtained by observing the foraging of other individuals to find food sources (Jones et al., 2018; Valone, 1989). However, group foraging can also have disadvantages, such as risk of kleptoparasitism, competition for the same prey and interference between predators reducing prey accessibility (Machovsky-Capuska et al., 2011a; Safina, 1990; Shealer and Burger, 1993). Alternatively, targeting prey that require less chase and handling time can increase efficiency as this increases the time and energy a predator has left to search for more prey. Chase and handling time can be influenced by the type and size of the prey, prey abundance and the presence of conspecifics and heterospecifics (Banbura et al., 1999; Bindoo and Aravindan, 1992; Neill and Cullen, 1974; Thiebault et al., 2016). In addition, prey chase and handling requires energy, which offsets the net energy gain from the prey item. Efficient predators, therefore, should target prey with low chase and handling times and high nutritional content.

As marine predators, seabirds have to hunt for prey in a highly spatially and temporally variable environment and forage over large areas (Weimerskirch, 2007). Consequently, investigating seabird interactions with prey is logistically challenging. With recent technological advances, the use of animal-borne video data loggers has made observing predator–prey interactions possible from the perspective of the predator (Thiebot et al., 2017; Tremblay et al., 2014; Watanabe and Takahashi, 2013; Wells et al., 2016). These interactions can help us to understand the choices made while foraging, and increase our knowledge on the foraging ecology of seabird species (Fauchald, 2009; Votier et al., 2013). In the face of the various threats seabirds are facing while foraging at sea, a good knowledge of foraging ecology can help us to predict the effect of different direct and indirect threats, and eventually contribute to the conservation and management of studied species (Croxall et al., 2012; Lewison et al., 2012).

The Australasian gannet is a large pelagic seabird species breeding on islands, coastal locations and artificial structures along the coast of south-eastern Australia and New Zealand (Norman et al., 1998). Gannets are visual predators and previous research has shown that heterospecific predators are often used as a cue to locate prey (Thiebault et al., 2014). As with other members of the Sulidae (gannets and boobies), Australasian gannets use a plunge-diving technique for foraging. Through the use of underwater wingbeats, they have the potential to extend a dive from a short V-shaped dive to a longer U-shaped dive (Machovsky-Capuska et al., 2011b). However, plunge diving also has a high energetic cost, so it is assumed that a high prey capture rate is necessary to compensate this (Green et al., 2009). The diet of Australasian gannets consists mainly of schooling pelagic species such as anchovy, pilchard and garfish. Other commonly targeted species are barracouta, jack mackerel, red mullet and squids (Bunce, 2001).

Some major Australasian gannet colonies in Australia are located in or near Bass Strait in south-eastern Australia, and it is estimated that 75% of the breeding seabirds in Australia are supported by Bass Strait and its environment (Norman et al., 1998; Ross et al., 1995). Due to climate change, south-eastern Australia is one of the fastest warming oceanic regions in the world (Poloczanska et al., 2012). Climate change can cause changes in species distributions and this can result in changing predator prey interactions and food web structures (Johnson et al., 2011; Perry et al., 2005; Wernberg et al., 2011). This impact is also expected for seabirds and for this reason; a deeper understanding of their foraging ecology can help in their conservation (Chambers et al., 2011).

Previous research into Australasian gannet foraging behaviour and efficiency has mainly focussed on search time through the combined use of accelerometers and GPS loggers (Angel et al., 2015a; Grémillet et al., 2016). However, little is known of the factors influencing capture success, prey chase and handling durations and the profitability of prey. This information is important for understanding how environmental variability may influence foraging efficiency and, ultimately, breeding success (Bunce et al., 2002; Chambers et al., 2011). Such knowledge can provide insights into foraging choices Australasian gannets make and can be used to predict the effects of both natural and anthropogenic influences on this ecologically and economically important species (Zeppel and Muloin, 2008)

The aims of this study, therefore, were to determine in Australasian gannets: (1) the factors influencing capture success; (2) the factors influencing chase and handling duration; and (3) the factors influencing profitability. The aims were addressed by concurrently tracking individuals with GPS and animal-borne video data loggers from which we derived information on foraging behaviour, prey type and density and the presence of con- and heterospecifics.

## RESULTS

### Prey types, foraging associations and factors influencing capture success

A total of 95 video data logger deployments, totalling 360 h, recorded 1031 prey encounter events, of which 594 were successful, 226 unsuccessful and 211 of unknown outcome (Table 1). The largest proportion of these dives (99%) were plunge dives. Ten ‘duck dives’ were observed, in which individuals dived to the shallow sea floor to hunt demersal prey from the surface (of which 50% were successful). Due to the rarity of this dive strategy, it was excluded from further analyses. For a high proportion of the dives the time spent underwater was short, with 58% of the dives lasting less than 5 s and 88% of the dives lasted less than 10 s. Only 12% of the dives lasted longer than 10 s, with the longest recorded dive lasting 36 s.

**Table 1.**
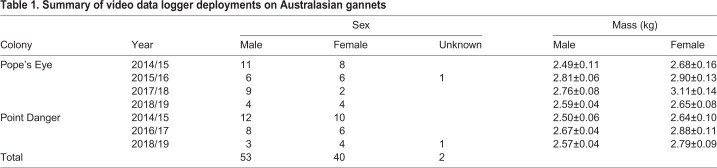
**Summary of video data logger deployments on Australasian gannets**

A large proportion of prey captures occurred outside the camera field of view, which resulted in a large number of prey events being classified as unknown (58% of the events). The highest proportions of identified prey were pilchard (*Sardinops sagax*) (21%), redbait (*Emmelichthys nitidus*) (3%) and anchovy (*Engraulis australis*) (3%) (Table 2). Juvenile Clupeiformes also contributed to a high proportion (4%) of the identified prey. However, all but one of these were captured by the same individual. Less abundant prey species were Gould's squid (*Nototodarus gouldi*) (1%), barracouta (*Thyrsites atun*) (1%) and red mullet (*Upeneichthys lineatus*) (1%), with the latter two species being consumed mostly by gannets foraging inshore in Port Philip Bay. Australian salmon garfish, jack mackerel and yellow-eye mullet were species observed rarely (<3 times each).

**Table 2.**
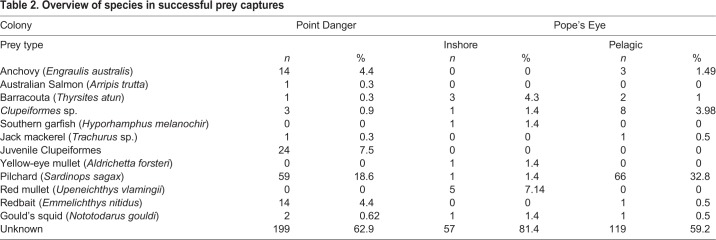
**Overview of species in successful prey captures**

In the inshore foraging strategy, most of the dives were solitary (79%) and conspecifics and heterospecifics were rare (21%). In contrast, in the pelagic strategy, solitary dives were less frequent (61%) and conspecifics and heterospecifics were more commonly observed during dives (39%) (Table 3). Conspecifics were observed for 33% of the dives. The most commonly observed heterospecifics were short-beaked common dolphins (*Delphinus delphis)* (16% of dives). While dolphins were often observed when in bait balls, gannets were also observed to be diving near dolphins that appeared to be in transit. Other commonly observed heterospecifics were Australian fur seals (*Arctocephalus pusillus doriferus*) (4%), shearwaters (*Ardenna* sp.) (5%), albatrosses (*Thalassarche* sp.) (2%) and sharks (*Elasmobranchii* sp.) (1%). Other species such as gulls (*Laridae* sp.), terns (*Thalasseus* sp.), little penguin (*Eudyptula minor*), and predatory fishes such as tuna (*Thunnus* sp.), were only observed in multi-species feeding aggregations.

**Table 3.**
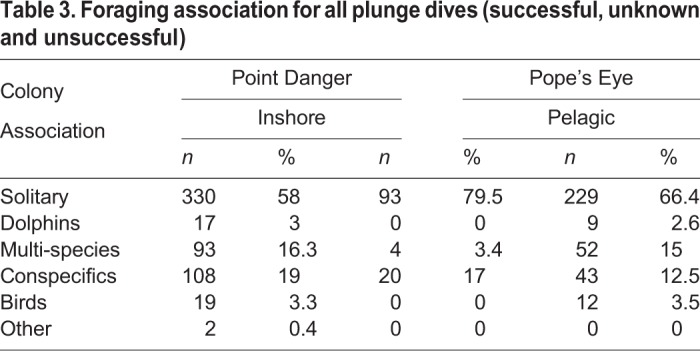
**Foraging association for all plunge dives (successful, unknown and unsuccessful)**

**Fig. 1.**
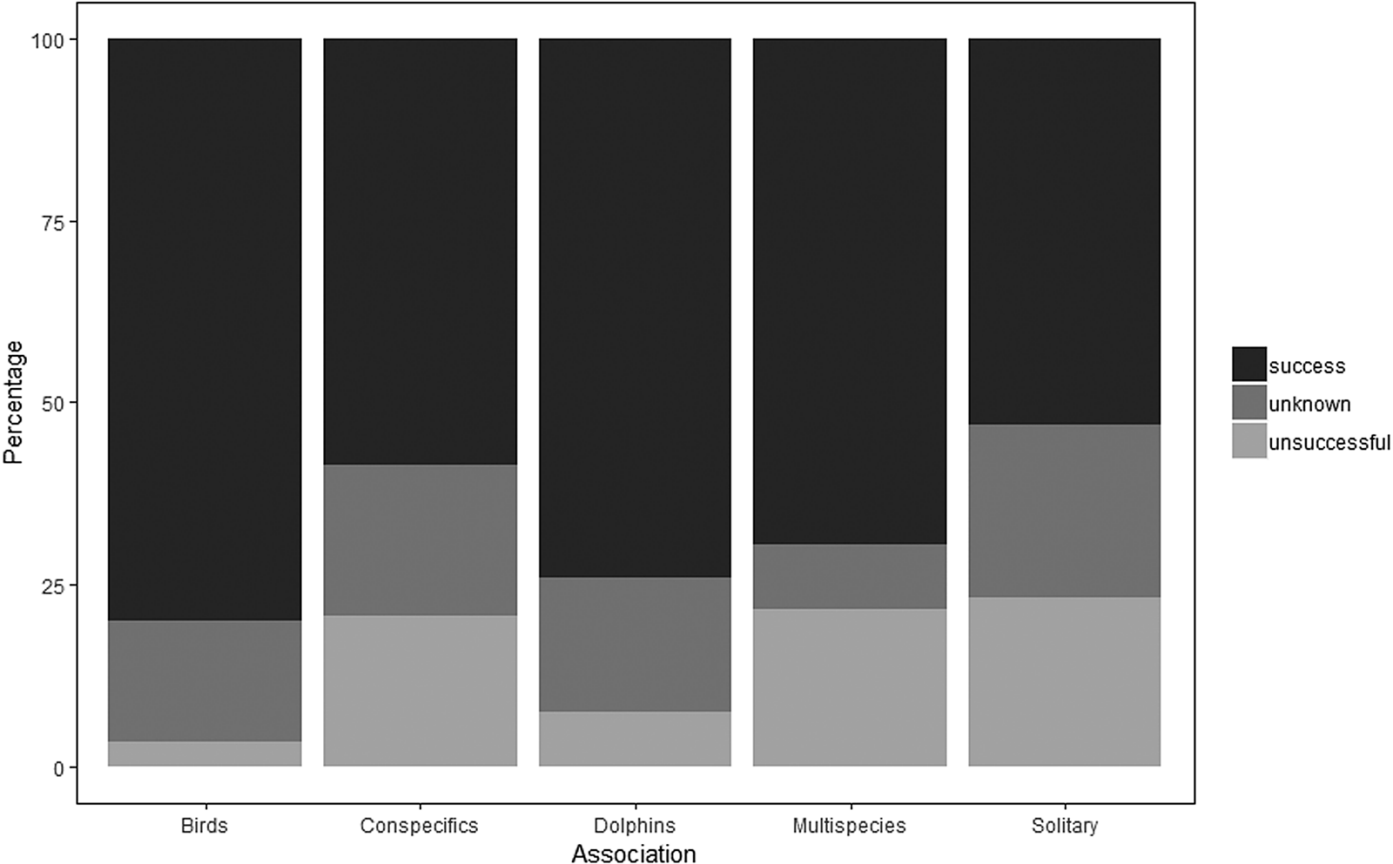
**Dive outcome for different associations.**

As no single model of the factors influencing capture success was clearly parsimonious (Table S2), model averaging was applied to obtain the effect of different parameters (Table S3), indicating that birds had a reduced capture success when foraging solitary [95%CI (−4.33, −0.29)] than when conspecifics or heterospecifics were present (Fig. 1). An additional model with association being either solitary or non-solitary also indicated that birds had a reduced capture success when foraging solitarily [95%CI (−0.83, −0.13)].

### Factors influencing chase duration, handling duration and profitability

As no single model was clearly parsimonious, model averaging was conducted for chase duration, handling duration and profitability analysis. For chase duration, the majority of candidate models included prey density and association, indicating that these factors are the most important to explain chase time (Table 4; Table S4). For foraging association, multi-species (presence of both conspecifics and marine mammals) was found to increase chase duration [95%CI (0.02, 1.02)]. The other factor influencing chase duration was the prey density, with a higher prey density increasing the chase duration [95%CI (0.06, 0.10)] (Table S5).

**Table 4.**
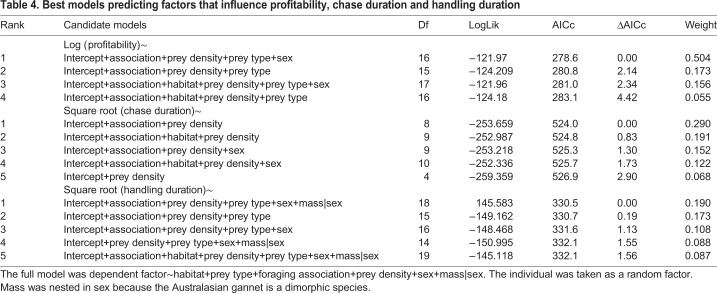
**Best models predicting factors that influence profitability, chase duration and handling duration**

All candidate models for handling duration included prey type and prey density but all models had small weights (Table 4; Table S6). Model averaging revealed prey density positively influenced handling duration [95%CI (0.02, 0.04)]. For prey type, only barracouta was observed to increase handling time [95%CI (0.79, 1.82)] (Table S7). The model for surface handling time had increased handling time for barracouta [95%CI (1.26, 2.07)] and squid [95%CI (0.14, 1.03)]. There was also a small effect of prey density [95%CI (0.005, 0.02)].

**Fig. 2.**
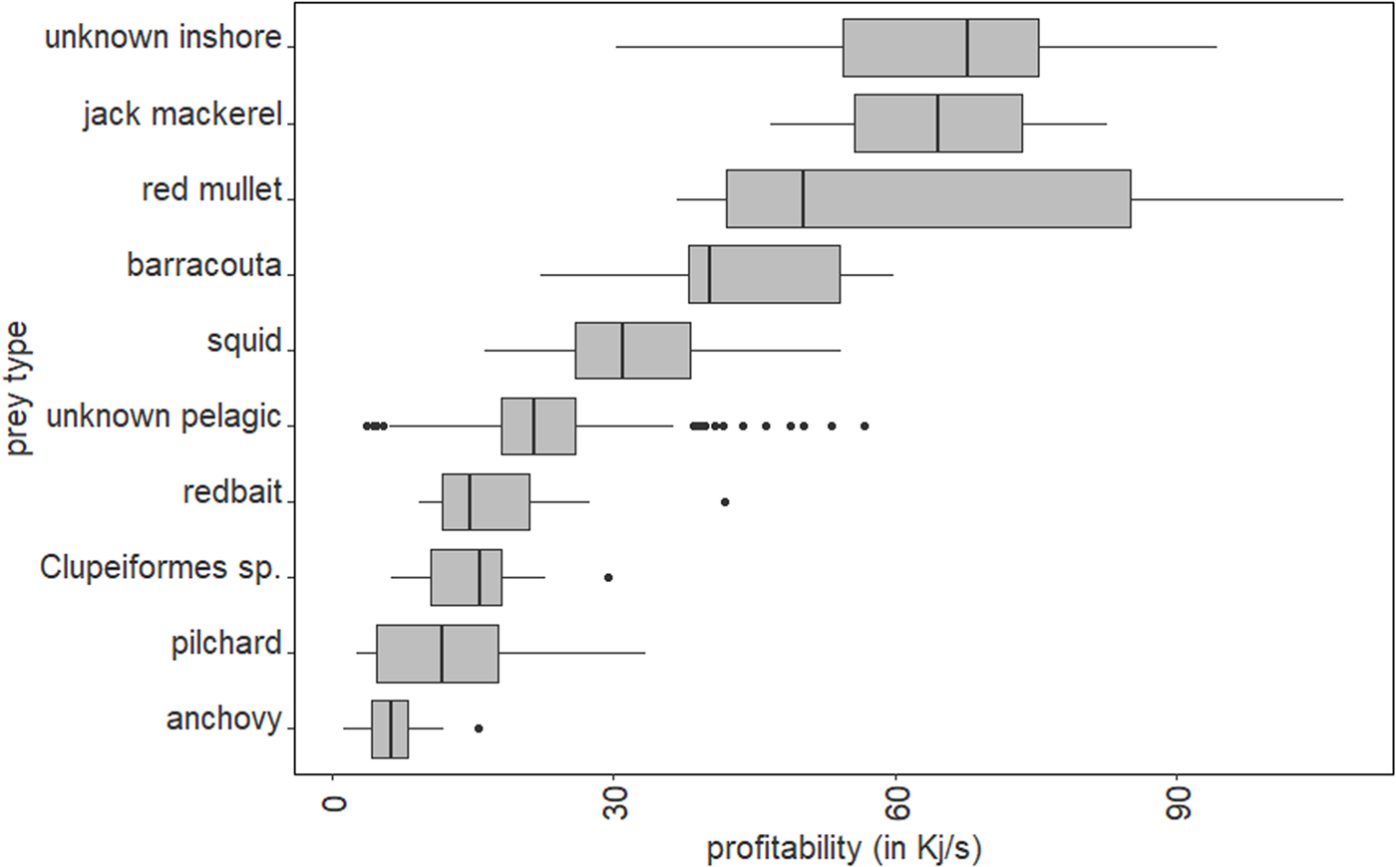
**Profitability for the different prey types.**

For profitability, numerous candidate models indicated that prey type, prey density and foraging association had an influence (Table 4). Prey density was found to have a negative influence on profitability [95%CI (−0.06, −0.04)]. Foraging in a multi-species association was found to have a negative influence on profitability [95%CI (−0.56, −0.09)] (Table S8). All prey types were found to have a difference in profitability (Fig. 2).

**Fig. 3.**
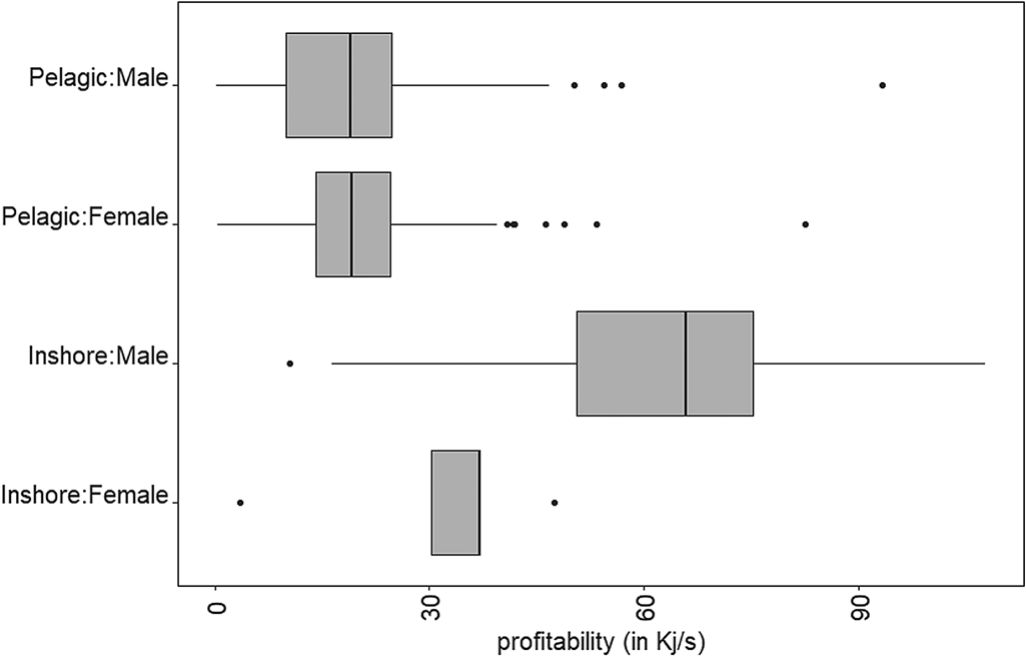
Profitability for the different sexes in the different habitats.

Sex and habitat are not significant variables, but as the available prey types in the inshore and pelagic habitat are different and the inshore strategy is mainly adopted by males, a difference in profitability can be expected for these factors. Within the same foraging strategy there was no observed difference between the sexes, but for both sexes profitability was generally higher inshore than in the pelagic habitat (Fig. 3). Only two females were observed to forage inshore, and the amounts of successful prey events for this group is limited, so this data should be interpreted with caution.

## DISCUSSION

Prey in the marine environment has an unpredictable and patchy distribution in both space and time, and studying the foraging behaviour of animals can assist in the understanding of how predators cope with this environmental variability (Thayer and Sydeman, 2007; Trathan et al., 2007). Previous research has suggested that individuals maximize their energy intake per time unit (Krebs et al., 1978) by reducing prey handling time and/or reducing search time (MacArthur and Pianka, 1966). While search time in foraging seabirds has been studied extensively (Garthe et al., 2014; Patrick et al., 2014; Weimerskirch et al., 1997), little is known about the factors influencing capture success and prey profitability. The use of bird-borne cameras in the present study enabled the factors influencing capture success, chase and handling time and prey profitability to be investigated. The results highlighted that multi-species foraging associations increase chase duration and decrease capture success and profitability. Prey density increases chase and handling duration and consequently decreases profitability. Prey type was of influence on both handling duration and profitability.

### Prey types, foraging associations and capture success

The prey identified in the present study are consistent with the previous reports of the Australasian gannet diet in the region (Bunce, 2001; Rodríguez Malagón, 2018; Schuckard et al., 2012). For a high proportion of dives, the prey type was unknown and as most of these dives were short dives, the data suggests that small schools or single prey were targeted in these cases.

Previous studies have found sociality in the foraging of gannets, and the use of conspecifics and heterospecifics as cues to locate prey (Jones et al., 2018; Thiebault et al., 2014; Tremblay et al., 2014). In the present study, individuals were often observed to forage with conspecifics. Gannets were also observed to forage near heterospecifics, of which dolphins were the most abundant. Interestingly, prey captures were in some cases observed near a group of dolphins that appeared to be in transit. In these instances, it is possible the dolphins were in the process of herding prey before the formation of a full bait ball. Multi-species foraging associations with a high abundance of conspecifics and heterospecifics were also observed frequently. The most abundant heterospecifics in these associations were dolphins, suggesting that they play an important role in increasing food accessibility for seabirds, as has been observed in other studies (Vaughn et al., 2008). In some cases, gannets were observed to be foraging on schooling prey with groups of shearwaters and albatrosses present, but no dolphins. It is possible that in these instances the avian predators continued to forage on the aggregated schooling prey after dolphins had left the area.

Despite the information source conspecifics can offer for locating prey (Thiebault et al., 2014), a large proportion of the dives in the present study were undertaken while individuals were solitary. For individuals foraging inshore, it might be expected that they would forage alone as most of the prey they encountered were non-schooling and, therefore, multiple captures in the same location are unlikely to be possible. These birds could be using private knowledge to locate these single prey types and avoid sharing it with other individuals (Milinski, 1994; Patrick et al., 2015; Votier et al., 2017; Wakefield et al., 2015). Previous research at the same study colony has shown a higher behavioural consistency in individuals foraging inshore than in pelagic habitat suggesting previous experience and knowledge may be the most important factors in locating prey in this habitat (Rodríguez Malagón, 2018).

The most important factor explaining success was feeding association, with solitary foraging being associated with the lowest probability of success. However, none of the factors explaining capture success had a high statistical weight in models, suggesting there are other important influential factors that were not assessed (e.g. prey type, prey density and individual age/experience). Due to the limitations of the study, these factors could not be included in the models and further research is required to properly assess their influence of prey capture success.

### Factors influencing chase, handling and profitability

Chase duration was most influenced by the type of foraging association and the prey density. In multi-species associations, increased dive duration was observed. This group contained all combinations of conspecifics, marine mammals and other heterospecifics. The fact that chase duration increased for this type of association could reflect that all predators present are competing for the same food source, leading to interference competition (Safina, 1990; Shealer and Burger, 1993). Indeed, in some videos conspecifics and heterospecifics could be observed targeting the same part of the bait ball, which could lead to interference competition.

Prey density was also observed to influence chase time, with a higher prey density increasing the chase time. This could be due to schooling by prey making it more difficult for the gannet to pick out a single prey item, thereby increasing the chase time. Relationships between chase time and school size have been observed for other predators (Neill and Cullen, 1974). Alternatively, the probability of a successful outcome when chasing prey may be greater for higher than lower prey densities and, therefore, individuals give up chases earlier when encountering low prey densities. Indeed, gannets can extend their dives by underwater wing flapping if the initial plunge dive is unsuccessful (Ropert-Coudert et al., 2009) but, as such activity is costly, they may only do so when the probability of capture success is high, which could be the case when prey density is high.

Prey density and prey type were found to be the most important factors influencing handling time in the present study. The effect of a higher prey density increasing the handling time may reflect individuals using underwater flapping to descend deeper when encountering high-density bait balls, leading to greater ascent durations once prey were captured. Demersal prey is only caught in shallow waters (Wells et al., 2016) and gannets likely dive deeper in bait balls than when hunting demersal prey. For prey type, only barracouta increased handling time significantly, which likely reflects the relatively large size of the prey captured and the time taken to manipulate it into position for swallowing. Similar long handling times were also observed for large prey such as jack mackerel and Australian salmon (excluded from models due to small sample size). In contrast, all smaller prey were swallowed quickly after surfacing such that there was a positive relationship between prey size and handling time, as has been observed in other species (Bindoo and Aravindan, 1992; Hoyle and Keast, 1987).

Prey density and prey type were also observed to influence the surface handling time, barracouta and squid were the only prey types that significantly increased surface handling time. Again this is likely due to the larger size and the time taken to manipulate the prey item for swallowing. A small effect of prey density was observed as well. This could indicate that prey in dense schools are slightly larger. However, as the observed effect size was very small, it is likely that the increase in time can be attributed to the fact that the exact time of swallowing was in some cases difficult to determine.

The results of the present study indicate prey profitability is influenced mostly by prey density, foraging association and prey type. Foraging in multi-species associations had a negative influence on prey profitability, due to the increased chase time. Similarly, the lower prey profitability at higher prey densities was due to increased chase and handling durations. The most profitable species had a higher energy content and a lower handling time, with species typically captured inshore (e.g. red mullet, barracouta) being more profitable than pelagic species (e.g. pilchard, anchovies and redbait).

Consequently, while habitat was not directly influencing profitability, inshore foraging was profitable due to the higher energy content of the prey species captured. In addition, factors associated to longer handling times, such as high prey density and multi-species foraging, only occurred in the pelagic habitat leading to higher prey profitability in inshore foraging. However, inshore foraging individuals are almost always solitary, whereas pelagic foragers can rely on social cues to locate food sources (Wells et al., 2016) and, when a multi-species association feeding on a bait ball is found, repeated dives are possible until the bird has captured enough prey or the bait ball breaks up (Thiebault et al., 2014). Hence, individuals may trade-off the costs of chase and handling time with search time in the different foraging strategies (MacArthur and Pianka, 1966). Interestingly, while capture success was lowest for inshore prey species, previous studies have found that individuals adopting an inshore foraging strategy make fewer dives than those in pelagic habitats (Rodríguez Malagón, 2018). This suggests that the higher profitability of inshore prey is sufficient to meet the nutritional needs of individuals despite their longer search time and lower capture success.

In summary, the results of the present study have documented differences in the chase duration, capture success, handling duration and profitability of prey consumed by Australasian gannets. These differences highlight the potential impacts of changes in the distribution and abundance of various prey species on Australasian gannets. Such impacts may have significant consequences for the population in view of its rapidly warming oceanic habitat (Poloczanska et al., 2012). As no factors stood out as a strong predictor of the variables of interest, other factors not considered here are also likely to be having an influence. Therefore, while this study found some factors of importance, these are likely only a limited part of the factors at play, and further research will be needed to get a more complete view of factors influencing gannet foraging. In addition, future research employing technological advances to obtain video data of complete foraging trips is needed to incorporate search time into the analyses of prey profitability in order to more fully understand the prey choices of the Australasian gannets.

## MATERIALS AND METHODS

### Study site and animal handling

**Fig. 4.**
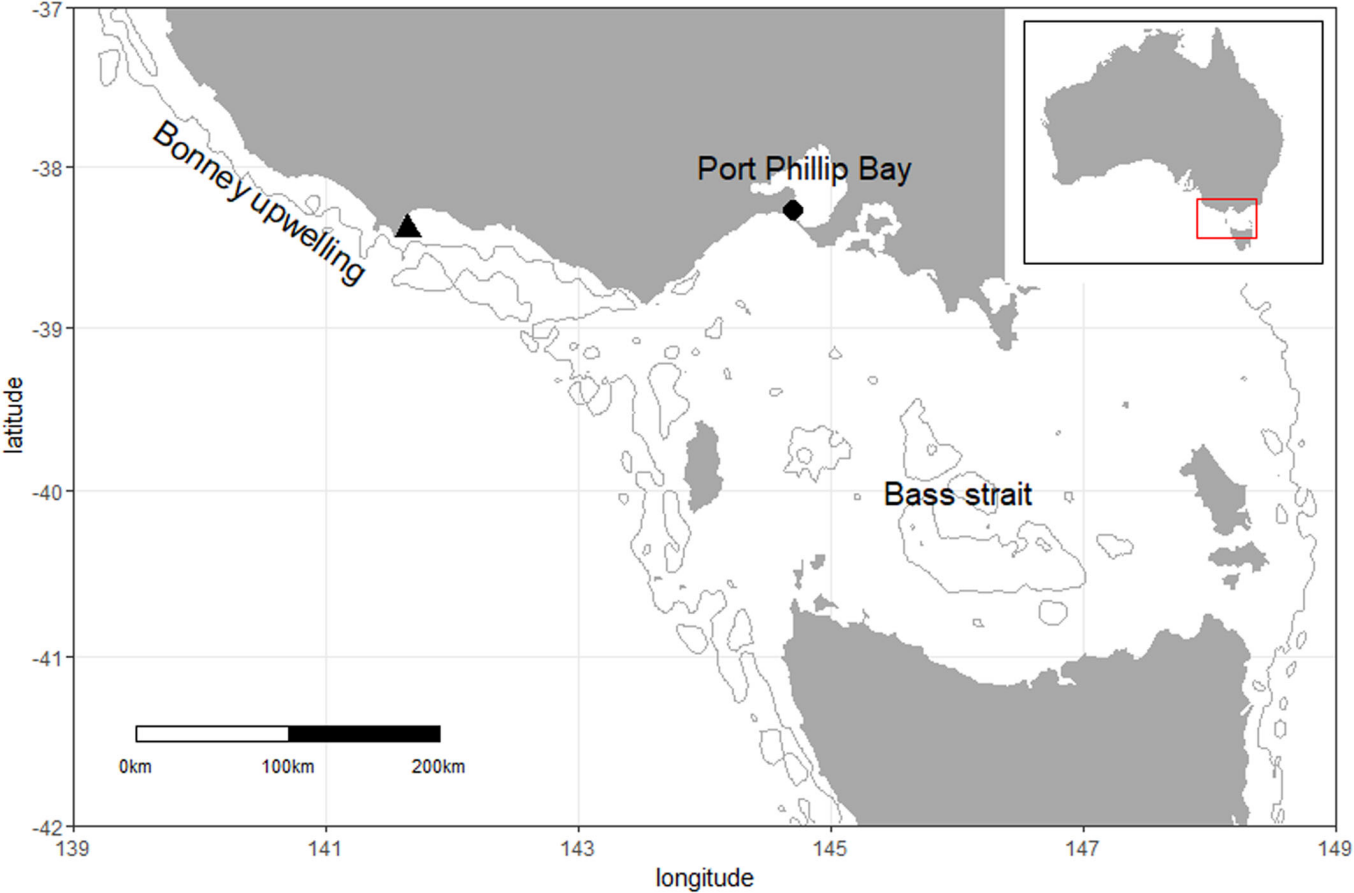
**Location of the study colonies.** Triangle: Point Danger colony; circle: Pope's Eye colony.

The study was conducted during the breeding seasons of 2014/15, 2016/17, 2017/18 and 2018/19 at Pope's Eye (38°16′42″S, 144°41′48″E) and 2014/15, 2017/18 and 2018/19 at Point Danger (38°23′36″S, 141°38′54″E; Fig. 4). The Point Danger colony (*ca* 300–400 nests) is the only mainland colony in Australia and is located near to the highly productive and nutrient-rich seasonal (November to April) Bonney Upwelling (Nieblas et al., 2009). The Pope's Eye colony (*ca* 180 nests) is located on an artificial structure close to the entrance of Port Philip Bay (Pyk et al., 2013), a large shallow bay with a maximum depth of 24 m. To the south of the Port Phillip Bay lies Bass Strait, the shallow (maximum depth 60–80 m) continental shelf area between mainland Australia and Tasmania with limited nutrient supply and high mixing (Gibbs et al., 1986). Previous research has shown that gannets from Pope's Eye forage both inside Port Philip Bay and in Bass Strait (Wells et al., 2016).

Breeding adults were captured at the nest with a noose pole (Point Danger) or by hand (Pope's Eye). To ensure the chick was not left unguarded and to minimise disturbance, the departing partner was captured during a changeover (Votier et al., 2013). Individuals were weighed in a cloth bag using a suspension scale (±25 g) and equipped with a GPS data logger (IgotU120, Mobile Action Technology, 44.5×28.5×13 mm, 12 g) and a miniature video data logger (Birdcam, Catnip Technologies, 25×45×15 mm, 24 g, 400×400 pixels at 28–30 frames per second, FOV 70°). In 2018/19, individuals were equipped with a combined GPS and tri-axial accelerometer data logger (X8 500mAph, Gulf Coast Data Concepts LLC, USA; 18 g) and video data logger. The video data loggers were programmed to record continuously in 2014/15 and with a 30 min on: 90 min off schedule in following breeding seasons. The devices were packaged in heat shrink tubing and attached with the camera pointing forward as a single unit to the central tail feathers with waterproof tape (Tesa^®^ 4651; Wilson et al., 1997).

A body feather was taken for genetic sexing and a uniquely numbered metal leg band was applied to the individual. Green waterproof marking paste was then applied to the neck of the bird for quick identification to facilitate recapture before the individual was released at the edge of the colony to resume normal behaviour. After one or more foraging trips, individuals were recaptured and the devices were removed by peeling the tape from the feathers.

All animal handling followed protocols approved by Deakin University Animal Ethics Committee (B20/2013 and B34/2016) and the Department of Sustainability and Environment (Victoria, Australia) Wildlife Research Permits (10006878 and 10008086).

### Data processing

The downloaded video data were manually analysed frame-by-frame using a classification software program (Solomon Coder, version: beta 17.03.22; Péter, 2011). Information categorised included: behavioural state; foraging activity; presence of conspecifics and heterospecifics; conspecific and heterospecific abundance; prey type; prey density and capture success. The behavioural state category consisted of flapping, gliding, resting on the sea surface, plunge diving, pursuit diving, at sea preening and at colony (Fig. S1). Foraging activity contained chase time and handling time (Fig. S2). Chase time was categorised as starting as soon as the bird entered the water and stopped at the moment of prey capture or when the bird gave up the chase and started returning to the surface.

Prey capture outcome was determined as successful where there was direct prey capture or an indication of capture (enlarged gular pouch or slightly open beak); unsuccessful, where none of these signs could be observed; and unknown, where the video data quality was insufficient to accurately determine the outcome (Fig. S3). In all 594 successful prey capture events, no individual was observed capturing more than one item and individuals observed capturing prey always passively ascended to the surface with the bill angled slightly down and partially open. This suggests that gannets do not swallow their prey while underwater and, therefore, handling time was considered as starting at the moment of prey capture and ceased when reaching the surface if the prey was not observed anymore after reaching the surface. In cases where the prey was still observed after reaching the surface, the handling time ceased when the last sign of the prey was observed.

**Fig. 5.**
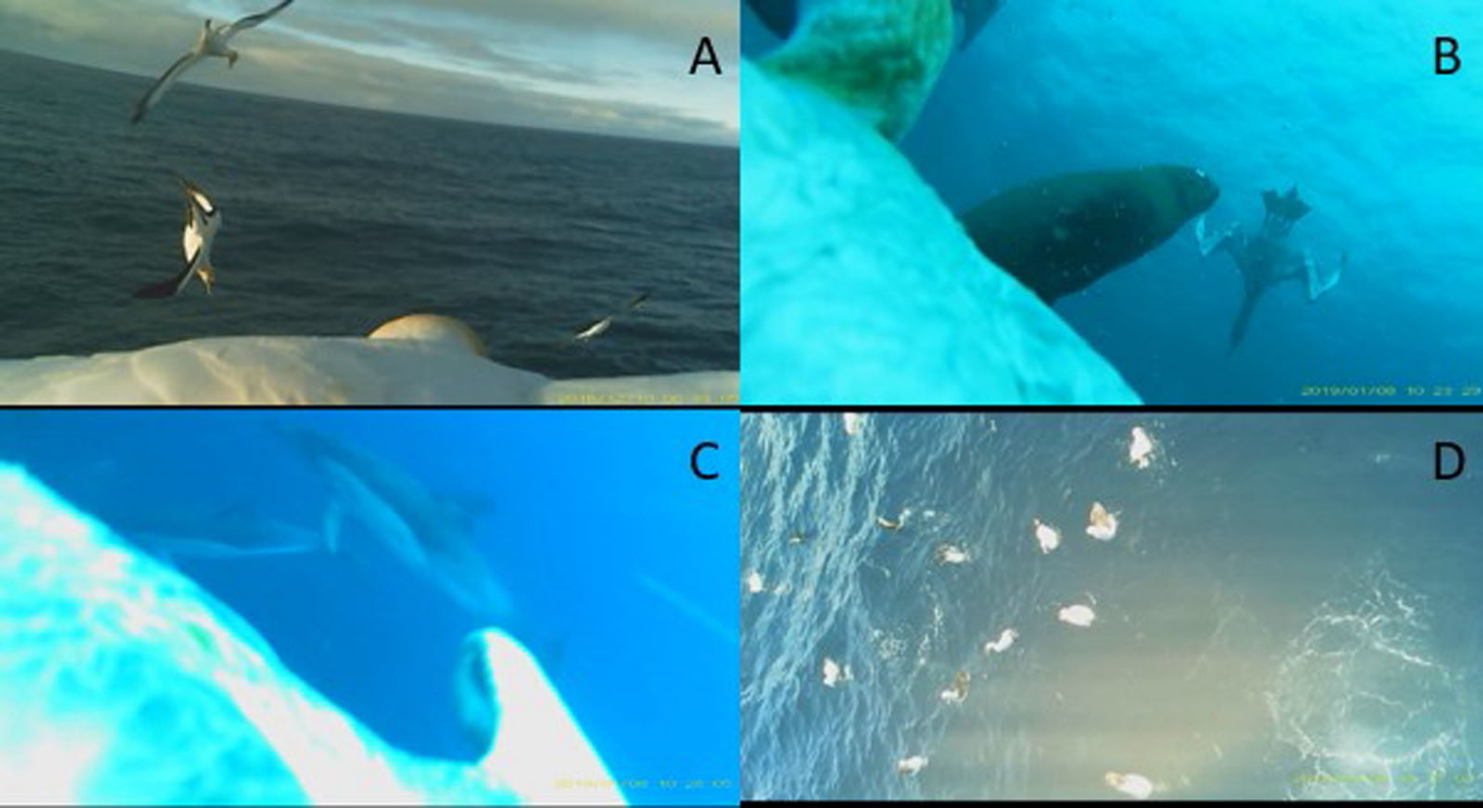
**Representative stills of observed foraging associations.** (A) Conspecifics (Australasian gannets), (B) multi-species (Australasian gannet and Australian fur seal), (C) dolphins (short-beaked common dolphins), (D) birds (albatrosses and shearwaters).

Presence of conspecifics and heterospecifics was also recorded for each dive. The presence of other gannets at or in the immediate surroundings of the dive location was recorded. Heterospecific species that were regularly observed at foraging locations were also recorded. These species were dolphins, fur seals, sharks, shearwaters and albatrosses. Since there was a high number of conspecific and heterospecific combinations observed, these were classified into association categories: solitary foraging (where no conspecifics or heterospecifics were observed); only conspecifics; only dolphins; multi-species (combination of conspecifics, mammals and other species); and birds (prey events with shearwaters and/or albatrosses present, but no mammals) (Fig. 5). When dives could not be placed in one of these classifications, they were placed in a miscellaneous category. Examples of these include a dive with only a fur seal present and a dive with only a shark present.

Fish were identified using a published identification guide (Gomon et al., 2008). Prey were not always observed in the camera field of view or were obstructed by the gannet. In such cases, prey were classified as unknown. To get an index of the density of prey at the foraging location, ten randomly selected frame images were obtained in which the prey species was clearly visible. In each of these frame images, a box square with sides of two fish fork lengths drawn randomly over the school and the number of fish in this area was counted. The average of these counts was then calculated to obtain a prey density index (PDI). In case a school was not observed during the dive, but the prey was identifiable, single prey or low prey abundance was assumed and the PDI was set as 1.

To estimate the energetic content of different prey types, information on their mass-specific energetic composition was obtained from the literature and combined with published estimates of the mass of prey captured by Australasian gannets (Table S1). As previous studies (Wells et al., 2016) have shown that individuals consume different prey depending on where they forage, information from the GPS data loggers was used to determine their foraging habitat [inshore (within Port Philip Bay); pelagic (out of Port Philip Bay)]. To estimate the energy content of unknown prey, the weighted average (by number of prey observations) of the energy content of the observed prey in the respective habitats was calculated and applied. This gave an energetic value of 332.18 kJ for unknown inshore prey and 119.62 kJ for unknown pelagic prey types. To estimate energy expenditure during prey chase and consumption, the activity specific heartrate for foraging in Australasian gannets was obtained (Green et al., 2009). Based on this heartrate VO_2_ consumption per minute for foraging was estimated (Green et al., 2013). The obtained VO_2_ consumption per minute was converted to energy expenditure using a value of 20.1kJ per litre O_2_ (Enstipp et al., 2006). This provided a coarse estimate of foraging cost of 59 J s^−1^. To calculate the profitability of prey (kJ s^−1^), the following equation was used:
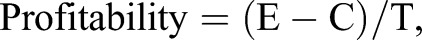
where E is the energy content of the prey, C is the foraging cost (59 J s^−1^ for the duration of chase time and handling time) and T is the total prey event duration.

### Statistical analyses

All statistical analyses were conducted in the R statistical environment (R Core Team, 2018). To analyse the factors contributing to success of the dive, only dives that were clearly identified as successful or unsuccessful were analysed using a generalized linear mixed model (GLMM) with a binomial distribution. The fixed factors in the full model contained foraging association, habitat (inshore-pelagic), sex and bird mass nested in sex as this species has been shown to be dimorphic at the study colonies (Angel et al., 2015b). To account for repeated measures, the individual was taken as a random factor.

For foraging association the ‘miscellaneous’ group was not included in the analysis. Prey type was not included in the model as this was unknown for most unsuccessful dives. Prey density was not included in the model as there was no prey observed in most unsuccessful dives, which also prevented obtaining good estimates. The dredge function of the *MuMin* package (Barton, 2018) was used for model averaging and comparison. The candidate models were ranked based on AICc and model averaging was applied for the models with ΔAICc<4. An additional model was made with the same parameters, but with association simplified to a solitary and a non-solitary group.

We ran a series of models to analyse factors influencing chase duration, handling duration, surface handling duration and profitability. The dependent factors chase, handling and surface handling duration were square root transformed. Profitability was log transformed. A linear mixed effects model based on maximum likelihood was used (Bates et al., 2015). The full model included habitat, prey type, prey density, foraging association and sex as fixed factors with bird mass nested in sex. Prey types for which there were less than three observations [garfish (*Hyporhamphus melanochir*), Australian salmon (*Arripis trutta*), jack mackerel (*Trachurus sp.*) and yellow-tailed mullet (*Aldrichetta forsteri*)] were excluded from the model. Juvenile Clupeiformes were also excluded as all but one of these were captured by the same individual. Observations for which prey type and density were both unknown were excluded from the model. Dives with foraging association ‘miscellaneous’ were also excluded. To account for repeated measures, the individual was taken as a random factor. To get an estimate of the effect of the parameters, the same model selection procedure as used for capture success was used. Residuals plots were visually inspected to assess normality and heteroscedasticity. Collinearity for the fixed continuous factors in the model was analysed by inspecting correlation coefficients in a Pearson correlation matrix and, where coefficients of *r*>0.7 were observed, one of the parameters was excluded from the model (Schober et al., 2018).

## Supplementary Material

Supplementary information
