# Supplementary material for: Factors influencing prey capture success and profitability in Australasian gannets (Morus serrator)
Source: Biol Open. 2020 Jan 24;9(1):bio047514. doi: 10.1242/bio.047514 (PMC6994950; doi:10.1242/bio.047514)
Supplement: Supplementary information [file biolopen-9-047514-s1.pdf]

Supplementary material

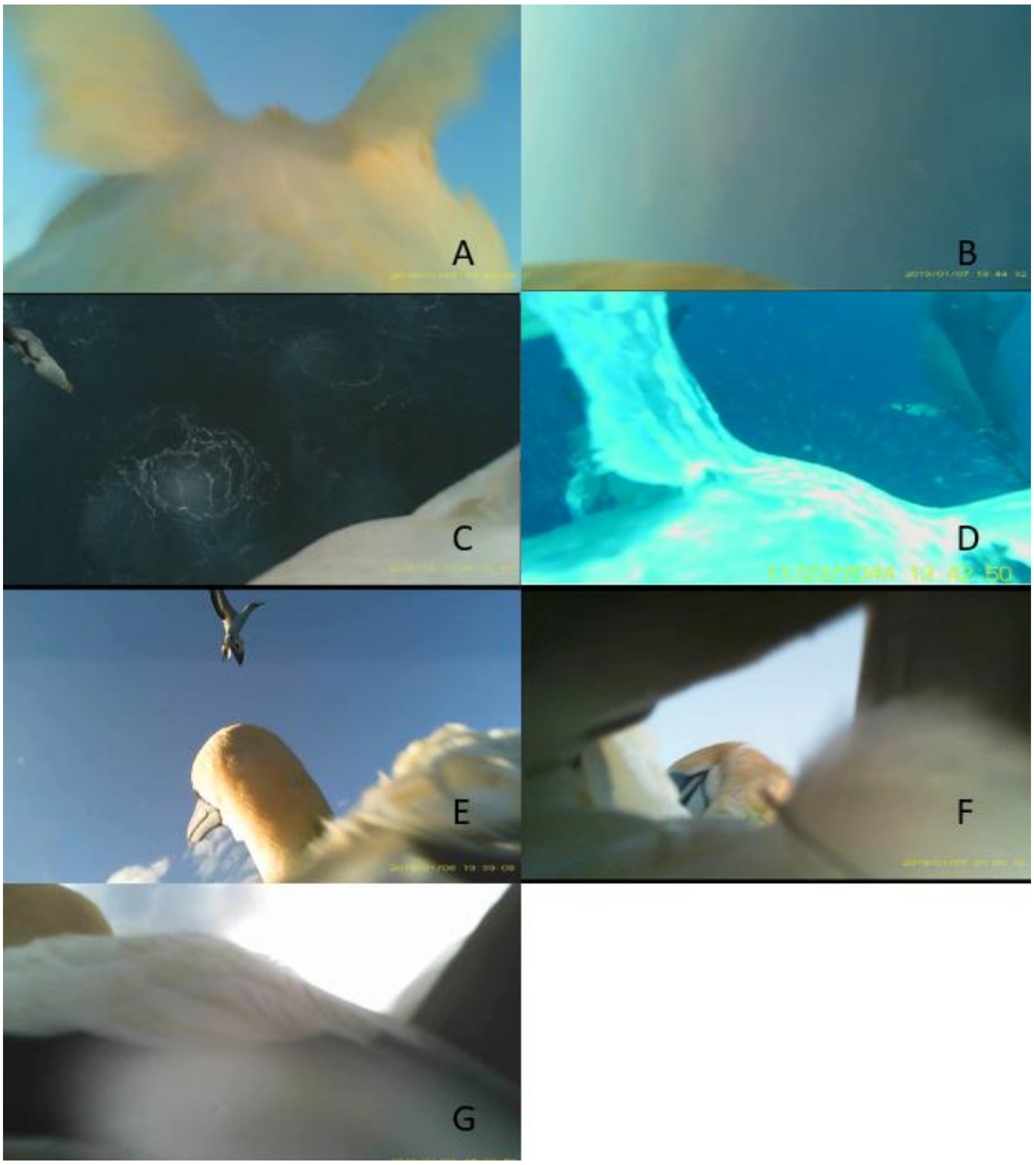

Figure S1: representative stills behavioural states: A)Flapping B)Gliding C)Plunge diving D)Pursuit diving E)Resting sea surface F)Preening G)At colony

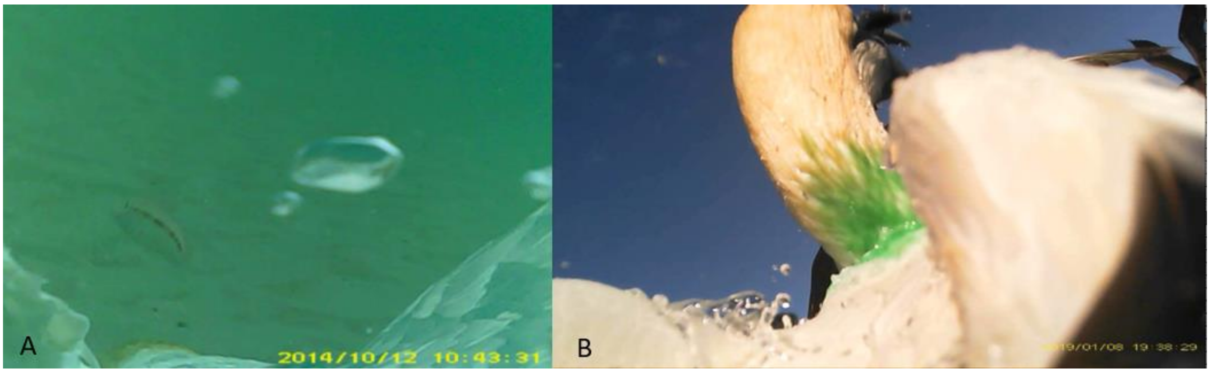

Figure S2: representative stills of foraging behaviour: A)chase B)handling

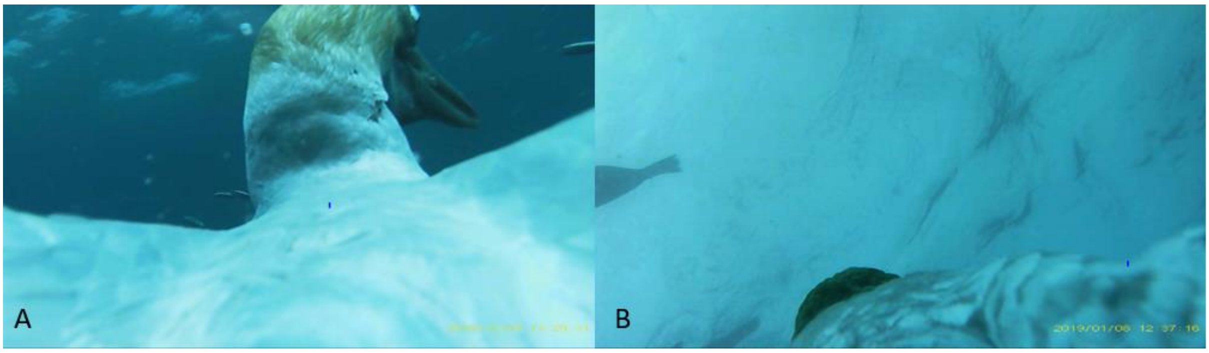

Figure S3: representative stills of dive success: A)Successful B)unsuccessful

Biology Open • Supplementary information

Table S1: Energy content for the different prey types encountered.

Species	Mean mass (g)	E content (kJ·g^{-1} wet mass)	Total E content (kJ)	Source
Anchovy (*Engraulis australis*)	9.85	5.2	51.27	1,2
Australian Salmon (*Arripis trutta*)	84.6	7.12	602.35	3,4
Barracouta (*Thyrsites atun*)	108.79	4.8	522.23	1,5
Clupeiformes sp.			97.56	
Southern garfish (*Hyporhamphus melanochir*)	42.05	5.7	239.68	1,2
Jack mackerel (*Trachurus* sp.)	163.84	5.65	925.73	1,6
Juvenile Clupeiformes	0.92	2.2	2.024	8
Yellow-eye mullet (*Aldrichetta forsteri*)	27.9	4.58	127.78	4,7,8
Pilchard (*Sardinops sagax*)	21.27	4.84	102.95	1,9
Red mullet (*Upeneichthys vlamingii*)	87.72	4.62	405.28	1,9
Redbait (*Emmelichthys nitidus*)	57.67	4.53	261.25	1,9
Gould's squid (*Nototodarus gouldi*)	44.81	4.7	245.59	1,10

Source: [1](Rodríguez Malagón, 2018); [2](Bunce, 2001); [3](Wells et al., 2016); [4](McCluskey et al., 2016); [5](Smith, 2011); [6](Balmelli and Wickens, 1994); [7](Schuckard et al., 2012); [8](Froese and Pauly, 02/2019); [9](Wiebkin, 2012); [10](Green et al., 1988)

Biology Open • Supplementary information

Table S2: best models predicting factors that influence prey event success. The full model was success ~ foraging association + habitat + sex + mass|sex. The individual was taken as a random factor. Mass was nested in sex because the Australasian gannet is a dimorphic species. The distribution was binomial.

Rank	Candidate models	Df	LogLik	AICc	ΔAICc	Weight
	Success~					
1	Intercept + association + sex + mass|sex	9	-447.559	913.3	0.00	0.316
2	Intercept + association	6	-450.775	913.7	0.31	0.271
3	Intercept + association + habitat + sex + mass|sex	10	-447.483	915.2	1.90	0.122
4	Intercept + association + sex	7	-450.583	915.3	1.96	0.119
5	Intercept + association + location	7	-450.705	915.6	2.20	0.105
6	Intercept + association + location + sex	8	-450.555	917.3	3.95	0.044
7	Intercept + sex + sex|mass	5	-455.031	920.1	6.79	0.011

Biology Open • Supplementary information

Table S3: Influence of parameters on capture success after model averaging of all models with ΔAICc<4 (full average)

	Estimate	SE	95% confidence interval	
			Lower limit	Upper limit
Intercept	2.51	1.61	-0.65	5.67
AssociationConspecifics	-2.02	1.04	-4.07	0.03
AssociationDolphins	-0.86	1.26	-3.35	1.62
AssociationMultispecies	-1.99	1.04	-4.04	0.05
AssociationNone	-2.31	1.03	-4.33	-0.29
SexM	-0.55	1.42	-3.36	2.24
Mass|SexFemale	0.23	0.42	-0.61	1.07
Mass|SexMale	0.49	0.63	-0.75	1.73
LocationPelagic	-0.02	0.15	-0.33	0.27

Biology Open • Supplementary information

Table S4: best models predicting factors that influence chase duration. The full model was square root(chase duration) ~ habitat + prey type + foraging association + prey density + sex + mass|sex. The individual was taken as a random factor. Mass was nested in sex because the Australasian gannet is a dimorphic species

Rank	Candidate models	Df	LogLik	AICc	ΔAICc	Weight
	Square root chase duration~					
1	Intercept + association + prey density	8	-253.659	524.0	0.00	0.290
2	Intercept + association + habitat + prey density	9	-252.987	524.8	0.83	0.191
3	Intercept + association + prey density + sex	9	-253.218	525.3	1.30	0.152
4	Intercept + association + habitat + prey density + sex	10	-252.336	525.7	1.73	0.122
5	Intercept + prey density	4	-259.359	526.9	2.90	0.068
6	Intercept + association + prey density + sex + mass|sex	11	-252.339	528.0	3.95	0.040
7	Intercept + association + habitat + prey density + sex + mass|sex	12	-251.399	528.3	4.31	0.034

Biology Open • Supplementary information

Table S5: Influence of parameters on square root (chase duration) after model averaging of all models with ΔAICc<4 (full average)

	Estimate	SE	95% confidence interval	
			Lower limit	Upper limit
Intercept	1.33	0.43	0.47	2.19
AssociationConspecifics	0.24	0.22	-0.19	0.68
AssociationDolphins	0.28	0.30	-0.31	0.87
AssociationMultispecies	0.52	0.25	0.02	1.02
AssociationNone	0.06	0.22	-0.37	0.49
Prey density	0.08	0.008	0.06	0.10
HabitatPelagic	-0.12	0.24	-0.59	0.34
SexMale	-0.07	0.32	-0.72	0.57
SexFemale|mass	-0.01	0.10	-0.23	0.19
SexMale|mass	-0.01	0.09	-0.20	0.18

Biology Open • Supplementary information

Table S6: best models predicting factors that influence handling duration. The full model was square root(handling duration) ~ habitat + prey type + foraging association + prey density + sex +mass|sex. The individual was taken as a random factor. Mass was nested in sex because the Australasian gannet is a dimorphic species

Rank	Candidate models	Df	LogLik	AICc	ΔAICc	Weight
	Square root(handling duration)~					
1	Intercept + association + prey density + prey type + sex + mass|sex	18	-145.538	330.5	0.00	0.190
2	Intercept + association + prey density + prey type	15	-149.162	330.7	0.19	0.173
3	Intercept + association + prey density + prey type + sex	16	-148.468	331.6	1.13	0.108
4	Intercept + prey density + prey type + sex + mass|sex	14	-150.995	332.1	1.55	0.088
5	Intercept + association + habitat + prey density + prey type + sex + mass|sex	19	-145.118	332.1	1.56	0.087
6	Intercept + association + habitat + prey density + prey type	16	-148.729	332.2	1.65	0.083
7	Intercept + prey density + prey type	11	-154.466	332.2	1.70	0.081
8	Intercept + association + habitat + prey density + prey type + sex	17	-148.089	333.2	2.73	0.049
9	Intercept + habitat + prey density + prey type + sex + mass|sex	15	-150.580	333.5	3.02	0.042
10	Intercept + habitat + prey density + prey type	12	-154.026	333.6	3.06	0.041
11	Intercept + prey density + prey type + sex	12	-154.071	333.7	3.15	0.039
12	Intercept + habitat + prey density + prey type + sex	13	-153.667	335.1	4.60	0.019

Table S7: Influence of parameters on square root(handling duration) after model averaging of all models with ΔAICc<4 (full average)

	Estimate	SE	95% confidence interval	
			Lower limit	Upper limit
Intercept	1.55	0.74	0.08	3.01
AssociationConspecifics	0.07	0.13	-0.18	0.32
AssociationDolphins	-0.07	0.17	-0.40	0.26
AssociationMultispecies	0.17	0.16	-0.14	0.48
AssociationNone	-0.05	0.13	-0.31	0.20
Prey density	0.03	0.006	0.02	0.04
PreytypeBarracouta	1.30	0.26	0.79	1.82
PreytypeClupeiformes sp.	-0.19	0.21	-0.61	0.22
PreytypePilchard	-0.20	0.15	-0.50	0.09
PreytypeRed mullet	-0.08	0.32	-0.71	0.55
PreytypeRedbait	-0.08	0.21	-0.50	0.34
PreytypeSquid	0.31	0.28	-0.25	0.87
PreytypeUnknown	-0.28	0.15	-0.59	0.03
SexMale	0.22	0.60	-0.97	1.41
Mass|sexMale	0.18	0.25	-0.31	0.68
Mass|sexFemale	0.08	0.17	-0.25	0.43
HabitatPelagic	0.06	0.17	-0.27	0.41

Table S8: Influence of parameters on log(profitability) after model averaging of all models with ΔAICc<4 (full average)

	Estimate	SE	95% confidence interval	
			Lower limit	Upper limit
Intercept	2.02	0.22	1.59	2.45
AssociationConspecifics	-0.17	0.12	-0.41	0.08
AssociationDolphins	-0.10	0.17	-0.44	0.22
AssociationMultispecies	-0.32	0.12	-0.56	-0.09
AssociationNone	0.01	0.13	-0.24	0.27
Prey density	-0.05	0.004	-0.06	-0.04
PreytypeBarracouta	1.61	0.22	1.17	2.04
PreytypeClupeiformes sp.	0.84	0.18	0.48	1.20
PreytypePilchard	0.93	0.13	0.66	1.20
PreytypeRed mullet	1.98	0.25	1.48	2.48
PreytypeRedbait	1.91	0.18	1.54	2.29
PreytypeSquid	1.36	0.25	0.87	1.85
PreytypeUnknown	1.16	0.14	0.89	1.45
SexMale	0.13	0.10	-0.05	0.33
HabitatPelagic	-0.004	0.10	-0.19	0.18
